# Activation of dopamine D1 receptor decreased NLRP3-mediated inflammation in intracerebral hemorrhage mice

**DOI:** 10.1186/s12974-017-1039-7

**Published:** 2018-01-04

**Authors:** Tian Wang, Derek Nowrangi, Lingyan Yu, Tai Lu, Jiping Tang, Bing Han, Yuxin Ding, Fenghua Fu, John H. Zhang

**Affiliations:** 1 Department of Anesthesiology and Physiology, School of Medicine, Loma Linda University, 11041 Campus St, Risley Hall, Room 219, Loma Linda, CA 92354 USA; 2 School of Pharmacy, Key Laboratory of Molecular Pharmacology and Drug Evaluation (Yantai University), Ministry of Education, Collaborative Innovation Center of Advanced Drug Delivery System and Biotech Drugs in Universities of Shandong, Yantai University, Yantai, 264005 Shandong People’s Republic of China; 3 School of Life Science, Yantai University, Yantai, 264005 Shandong People’s Republic of China

**Keywords:** D1 receptor, Interferon beta, Intracerebral hemorrhage, Neuroinflammation, NLRP3

## Abstract

**Background:**

Inflammasomes are involved in diverse inflammatory diseases. Previous study reported that the neurotransmitter dopamine inhibited NLRP3 inflammasome activation via dopamine D1 receptor (DRD1). The present study aims to investigate the role of DRD1 on neuroinflammation in intracerebral hemorrhage (ICH) mice and the potential mechanism mediated by NLRP3 inhibition.

**Methods:**

One hundred and six male CD-1 mice were subjected to intrastriatal injection of bacterial collagenase or PBS. A68930 (DRD1 specific agonist) was administered by subcutaneous injection at 1 h after collagenase injection. Behavioral deficits and brain water content were assayed. The expression of Iba 1 and MPO levels were measured by immunofluorescence staining. The expressions of proteins in the DRD1/interferon-beta (IFN-beta)/NLRP3 signaling pathway were evaluated by western blotting.

**Results:**

Activation of the DRD1 by A68930 decreased brain edema and improved behavior at 24 and 72 h of ICH. A68930 inhibited partly the activation of microglia and the neutrophil infiltration after 24 h of ICH. IFN-beta, p-STAT1 increased while NLRP3, caspase 1, and IL-1beta decreased after A68930 administration in ICH mice. DRD1 antagonist and IFN-beta siRNA reversed effects of A68930 on neurological outcome and brain edema. DRD1 antagonist and IFN-beta siRNA blocked not only A68930-mediated increases of IFN-beta, p-STAT1 but also A68930-mediated decreases of NLRP3, caspase 1, and IL-1beta.

**Conclusions:**

DRD1 activation by A68930 improves neurological outcome through inhibition of NLRP3-mediated inflammation in ICH mice.

**Electronic supplementary material:**

The online version of this article (10.1186/s12974-017-1039-7) contains supplementary material, which is available to authorized users.

## Background

Intracerebral hemorrhage (ICH) is a devastating subtype of stroke and accounts for 10–15% of all stroke cases [1]. The overall mortality rate is about 40% by 1 month, and many survivors of ICH patients become severely disabled [2]. The primary brain damage occurs within minutes to hours after the onset of ICH, and during this time, the hematoma causes local tissue destruction. Then, cellular debris and breakdown of blood components initiate a secondary injury phase, which can last for days to weeks in the perihematoma region adjacent to the hematoma [3].

Inflammation is an important host defense response to brain injury after ICH. When ICH occurs, blood components enter the cerebral parenchyma. The inflammatory response begins immediately after the presence of blood components in the parenchyma, and is characterized by accumulation and activation of inflammatory cells. The resident microglia and astrocytes are believed to be the early inflammatory cells in response to the extravascular blood components [4]. The inflammatory process following by microglia activation involves an infiltration of various circulating inflammatory cells, including neutrophil, leukocyte, and macrophage. Subsequently, activated inflammatory cells release a variety of cytokines, chemokines, free radicals, and other potentially toxic chemicals [5]. As ICH processes, more cytotoxic substances such as hemoglobin, heme, and iron are released, leading to a new phase of inflammatory response. ICH-induced cell death leads to neutrophil and leukocyte infiltration into the brain, which further aggravates inflammatory injury [6].

Dopamine not only affects behavior, movement, endocrine, cardiovascular, renal, and gastrointestinal functions, but also regulates the immune systems [7]. Dopamine receptors are expressed in almost all immune cells. Activation of its receptors with dopamine or agonists has been reported to modulate the activation, proliferation, and cytokine production in immune cells [8]. In addition, dopamine D2 receptor (DRD2) may have anti-inflammatory effects after ICH and DRD2 agonists inhibit neuroinflammation and attenuate brain injury after ICH [9].

The NLRP3 inflammasome activation promotes the maturation and the release of several proinflammatory cytokines, such as interleukin-1beta (IL-1beta). Therefore, NLRP3 inflammasome plays critical roles in the initiation of inflammation and the development of immune responses [10]. Previous study indicates that dopamine inhibits NLRP3 inflammasome activation via dopamine D1 receptor (DRD1) [11]. A study demonstrates that IFN-beta also represses the NLRP3 inflammasome, possibly by increasing the phosphorylation of STAT1, thereby suppressing caspase-1-dependent IL-1beta maturation [12].

In this study, we used collagenase-induced ICH mice model to investigate whether A68930 (DRD1 specific agonist, Sigma) can improve neurological outcome through inhibition of NLRP3-mediated inflammation.

## Methods

### Animals

A total of 106 (excluding 3 mice that died because of anesthetic overdose) male CD1 mice (Charles River, Wilmington, MA, USA) weighing 30 to 34 g were used in these experiments. Animals were housed and maintained on a 12-h light/dark cycle at a controlled temperature and humidity with unlimited access to food and water. All animal experiments were approved by the Institutional Animal Care and Use Committee at Loma Linda University. The study followed the Guide for the Care and the Use of Laboratory Animals (National Research Council) and complied with the ARRIVE guidelines for reporting in vivo experiments.

### ICH model

ICH mouse model was induced as previously described [13]. Briefly, animals were intraperitoneally injected with ketamine (90 mg/kg) and xylazine (5 mg/kg). Rectal temperature was maintained at 37.5 °C with a heating pad. Then, the mice were positioned in a stereotaxic frame (Model 500, Kopf Instruments, Tujunga, CA, USA), and a hole (1 mm) on mouse skull was drilled near the right coronal suture 2.2 mm lateral to the midline. A 26-gauge needle was inserted stereotaxically into the right basal ganglia (coordinates 0.2 mm anterior, 3.5 mm ventral, and 2.2 mm lateral to the bregma). Bacterial collagenase (0.075 U dissolved in 0.5 μL of PBS) was infused at 0.167 μL/min by a microinfusion pump (Harvard Apparatus Inc., South Natick, MA, USA). After injection, the needle remained in the position for 5 min to prevent reflux and then it was gently removed. The hole was filled with bone wax, and the skin incision was closed with suture. Animals in sham group were subjected to the same operative procedure except were infused with PBS (0.5 μL). Then the ICH mice were randomly divided into vehicle or treatment groups by the experimenter who prepared the ICH models.

### Garcia test

Garcia test with a 21-point score was performed by another experimenter who was blinded to the experimental design. The assessment evaluating spontaneous activity, axial sensation, vibrissae proprioception, symmetry of limb movement, lateral turning, forelimb walking, climbing, and grabbing was conducted [14].

### Forelimb placing test

Forelimb placing test was investigated according to the previous method with minor modification [15]. Briefly, animals were positioned parallel to a table top and were slowly moved up and down, allowing the vibrissae on one side of the head to brush along the table surface. Refractory placement of the left and right forelimbs was investigated for 10 consecutive trials. Then, left forelimb placement was calculated as left forelimb placement/(left forelimb placement + right forelimb placement) × 100%.

### Brain water content

Brain water content was measured with the wet weight/dry weight method as previously described [16]. Mice under deep isoflurane anesthesia were decapitated at 24 or 72 h after surgery, and brains were quickly removed. Coronal sections were separated 2 mm anterior and posterior of the needle tract. These sections were further divided into the ipsilateral cortex (Ipsi-CX), contralateral cortex (Cont-CX), ipsilateral basal ganglia (Ipsi-BG), and contralateral basal ganglia (Cont-BG). The cerebellum was collected as an internal control. All brain samples were weighed using an analytical microbalance (APX-60, Denver Instrument, Bohemia, NY, USA). The samples were dried at 100 °C for 24 h before determining the dry weight. Brain water content (%) was calculated as (wet weight–dry weight)/wet weight × 100%.

### Immunofluorescence staining

Immunofluorescence staining was performed according to previous study [17]. A series of coronal brain sections (10 μm thick) were blocked in 5% bovine serum albumin for 2 h at room temperature and then were incubated with rabbit anti-DRD1 (1:200, Abcam), rabbit anti-IFN-beta receptor (1:50, Santa Cruz), goat anti-ionized calcium-binding adapter molecule 1 (Iba 1, 1:200, Abcam), and rabbit anti-MPO (1:100, Abcam) at 4 °C overnight. After being washed three times with PBS, the sections were incubated with appropriate fluorescence-conjugated secondary antibodies (1:300, Jackson ImmunoResearch) for 2 h at room temperature. The sections were rinsed three times for 5 min each with PBS. The loci around hematoma (black triangle, Fig. 4a) of stained sections were examined with a fluorescence microscope (Leica DMi8). Microphotographs were analyzed with LASX software.

### Western blotting

The proteins of ipsilateral/right hemispheres were extracted by cytoplasmic extraction reagents (Pierce Biotechnology, Rockford, IL, USA). Proteins were then loaded (50 μg) and separated by SDS-PAGE gel electrophoresis. After blocking with 5% nonfat milk for 1.5 h, the membranes were incubated overnight at 4 °C with the primary antibodies: rabbit anti-DRD1 (1:1000, Abcam), goat anti-IFN-beta (1:300, Santa Cruz), rabbit anti-IFN-beta receptor (1:500, Santa Cruz), rabbit anti-STAT1(1:2000, Cell Signaling), goat anti-p-STAT1 (1:500, Santa Cruz), rabbit anti-NLRP3 (1:1000, MyBioSource), rabbit anti-caspase1 (1:1000, NOVUS Biologicals), rabbit anti-IL-1beta (1:2000, Cell Signaling), and rabbit anti-β-actin (1:2000, Santa Cruz). β-actin served as the loading control. The membranes were processed with the respective horseradish peroxidase-labeled secondary antibody (Santa Cruz Biotechnology). Bands were visualized using the ECL detection reagents (Amersham Biosciences). The relative density of protein was analyzed by ImageJ software.

### Experimental design

The experiments were conducted as follows (Additional file 1: Figure S1).

#### Experiment 1

The time course of DRD1 and IFN-beta expressions after ICH were analyzed by western blotting method. The ipsilateral hemispheres of three animals in each group were collected at 3, 6, 12, 24, and 72 h after ICH. The ipsilateral hemispheres of three animals in sham group were also harvested.

#### Experiment 2

Mice were randomly divided into five groups with eight animals in each group: sham, ICH + vehicle, ICH + A68930 (1.0 mg/kg), ICH + A68930 (2.0 mg/kg), and ICH + A68930 (4.0 mg/kg). A68930 was administered by subcutaneous injection at 1 h after ICH, whereas the mice in sham and vehicle were subcutaneously injected with sterile saline. Neurological outcome (Garcia test and forelimb placement test) and brain water content were evaluated at 24 h after ICH. DRD1, IFN-beta, IFN-beta receptor, STAT1, p-STAT1, NLRP3, pro-caspase1, caspase1, pro-IL-1beta, and IL-1beta expressions in ipsilateral hemispheres were assayed at 24 h after ICH.

#### Experiment 3

Mice were randomly divided into three groups with five animals in each group: sham, ICH + vehicle, and ICH + A68930 (4.0 mg/kg). A68930 was administered by subcutaneous injection at 1 h after ICH, whereas the mice in sham and vehicle were subcutaneously injected with sterile saline. Neurological outcome (Garcia test and forelimb placement test) and brain water content were evaluated at 72 h after ICH.

#### Experiment 4

Animals were randomly divided into six groups with three mice in sham, vehicle, and A68930 (4.0 mg/kg) groups and eight mice in SCH23390 group, scramble siRNA group, and IFN-beta siRNA group. Scramble siRNA (100 pmol/2 μl, ORIGENE) or IFN-beta siRNA (100 pmol/2 μl, ORIGENE) were intracerebroventricularly injected 48 h before ICH model preparation in scramble siRNA group or IFN-beta siRNA group, respectively. SCH23390 (DRD1 specific antagonist, Sigma) at dose of 1.0 mg/kg was intraperitoneally given 30 min after collagenase injection in SCH23390 group. A68930 was administered by subcutaneous injection at 1 h after ICH in A68930 (4.0 mg/kg), SCH23390, scramble siRNA, and IFN-beta siRNA groups. Garcia test and forelimb placement test were performed at 24 h after ICH. Then the brain water content was evaluated. Western blotting of ipsilateral hemispheres was conducted at 24 h after ICH in all groups. The data of Garcia test, forelimb placement test, brain water content and the samples of ipsilateral hemispheres in sham, vehicle, and A68930 (4.0 mg/kg) groups were shared with experiment 2. Three mice in sham, vehicle, and A68930 (4.0 mg/kg) groups were performed with double immunofluorescence staining of Iba 1 and DRD1, Iba 1 and IFN-beta receptor. Furthermore, the immunofluorescence staining of Iba 1 and MPO in sham, vehicle, and A68930 (4.0 mg/kg) groups was also conducted.

### Statistical analysis

All data were expressed as mean ± SEM. The animals that died were excluded in the analyses. Analysis was performed using GraphPad Prism software 6.0. The data were analyzed with one-way ANOVA followed by Tukey’s post hoc test. Kruskal-Wallis one-way ANOVA analysis followed by Student-Newman-Keuls test was used for non-parametric comparison of neurological deficit scores. Statistical significance was defined as *p* < 0.05. A power analysis was performed using SAS 9.4 software. On the basis of the pilot preliminary study for neurobehavioral function, five mice were required to achieve 90% power to detect a difference, with a probability of *p* < 0.05.

## Results

### Expressions of DRD1 and IFN-beta after ICH

**Fig. 1:**
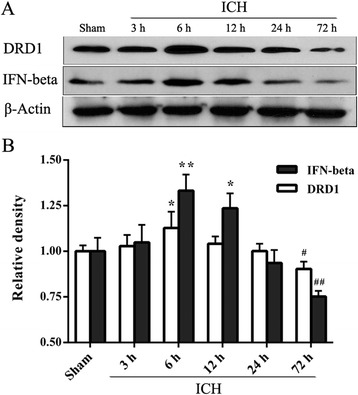
Expressions of DRD1 and IFN-beta in the ipsilateral hemispheres after intracerebral hemorrhage (ICH). **a** Representative photographs of DRD1 and IFN-beta in western blotting. **b** Bar graphs of quantitative analysis of DRD1 and IFN-beta expressions from the ipsilateral hemisphere after ICH. DRD1 and IFN-beta expressions increased, ^*^p < 0.05, ^**^p < 0.01 versus sham; DRD1 and IFN-beta expressions decreased, ^#^*p* < 0.05, ^##^*p* < 0.01 versus sham

Western blotting results showed that the expression of DRD1 increased at 6 h and decreased at 72 h after ICH (*p* < 0.05 versus sham). An elevation of IFN-beta in the ipsilateral hemispheres at 6 and 12 h after ICH was observed (*p* < 0.05 or *p* < 0.01 versus sham). The level of IFN-beta in the ipsilateral hemispheres also decreased at 72 h after ICH (*p* < 0.01 versus sham; Fig. 1).

### Effects of A68930 on neurological outcome and brain water content after ICH

**Fig. 2:**
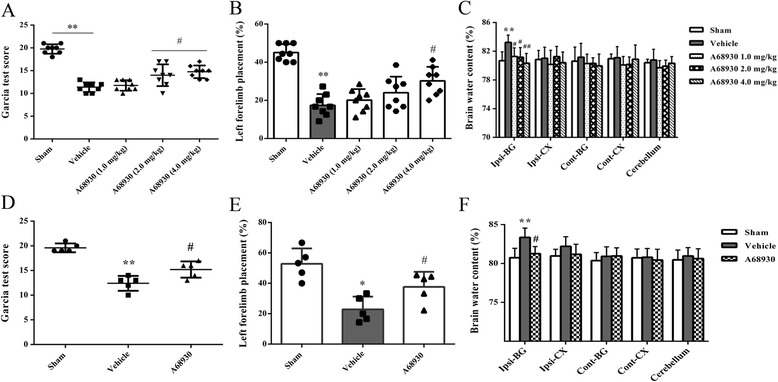
Effects of A68930 on neurological outcome and brain water content after intracerebral hemorrhage (ICH). Garcia test score (**a**), left forelimb placement (**b**), and brain water content (**c**) at 24 h after ICH. Garcia test score (**d**), left forelimb placement (**e**), and brain water content (**f**) at 72 h after ICH. ^*^p < 0.05, ^**^p < 0.01 versus sham; ^#^*p* < 0.05, ^##^*p* < 0.01 versus vehicle

At 24 and 72 h after ICH, mice in vehicle group showed worse performances in Garcia test (*p* < 0.01 versus sham; Fig. 2a, d) and limb placement test (*p* < 0.05 or *p* < 0.01 versus sham; Fig. 2b, e), as well as an increase of perihematomal brain water content in the ipsilateral basal ganglia (*p* < 0.01 versus sham; Fig. 2c, f). However, activation of the DRD1 receptor using high dose of A68930, the Garcia test and left forelimb placement scores were significantly improved (*p* < 0.05 versus vehicle). A68930 treatment also reduced the perihematomal brain edema (*p* < 0.05 or *p* < 0.01 versus vehicle).

### Effects of A68930 on the number of positive cells of MPO or Iba 1 after ICH

**Fig. 3:**
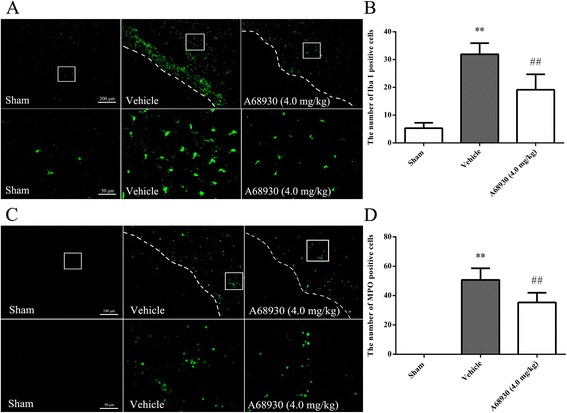
Effects of A68930 on the number of positive cells of Iba 1 or MPO after intracerebral hemorrhage (ICH). **a** Representative photographs of the Iba 1 positive cells in the perihematoma brain tissue. **b** Bar graphs of quantitative analysis of the Iba 1 positive cells in the perihematoma brain tissue. **c** Representative photographs of the MPO positive cells in the perihematoma brain tissue. **d** Bar graphs of quantitative analysis of the MPO positive cells in the perihematoma brain tissue. ^**^p < 0.01 versus sham; ^##^*p* < 0.01 versus vehicle

Immunofluorescence staining of MPO or Iba 1 was performed in sham, vehicle, and A68930 (4.0 mg/kg) groups at 24 h after ICH. The total number of positive cells of MPO or Iba 1 was significantly increased in vehicle group (*p* < 0.01 versus sham; Fig. 3). A68930 treatment inhibited partly the augment of Iba 1 or MPO positive cells in the perihematoma brain tissue (*p* < 0.01 versus vehicle).

### Expressions of DRD1 and IFN-beta receptor in microglia

**Fig. 4:**
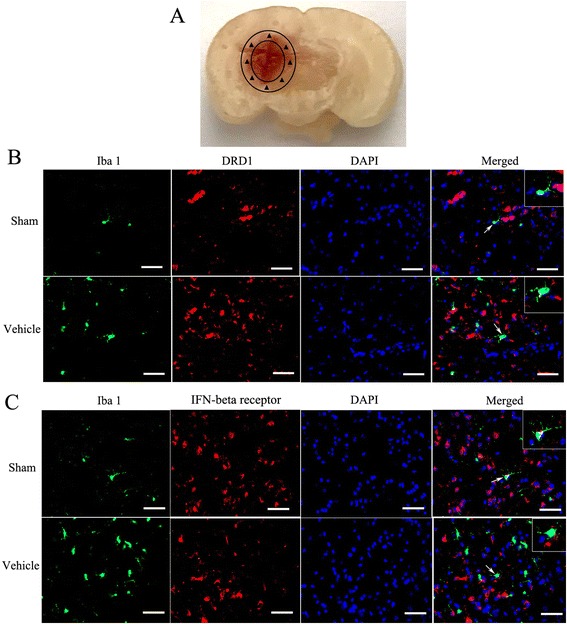
Expressions of DRD1 and IFN-beta receptor in microglia. **a** Co-localization of DRD1 with Iba 1 and of IFN-beta receptor with Iba 1 was observed in the perihematoma brain tissue (showing with black triangle) at 24 h after ICH. **b** Representative photographs of co-localization of DRD1 with Iba 1 (bar = 50 μm). **c** Representative photographs of co-localization of IFN-beta receptor with Iba 1 (bar = 50 μm)

Double immunofluorescence staining was performed in the perihematoma brain tissue of sham and vehicle groups. The staining found that both DRD1 and IFN-beta were co-localized with microglia (Iba 1 positive cells; Fig. 4).

### Effects of A68930 on the expressions of proteins in DRD1/IFN-beta/NLRP3 pathway after ICH

**Fig. 5:**
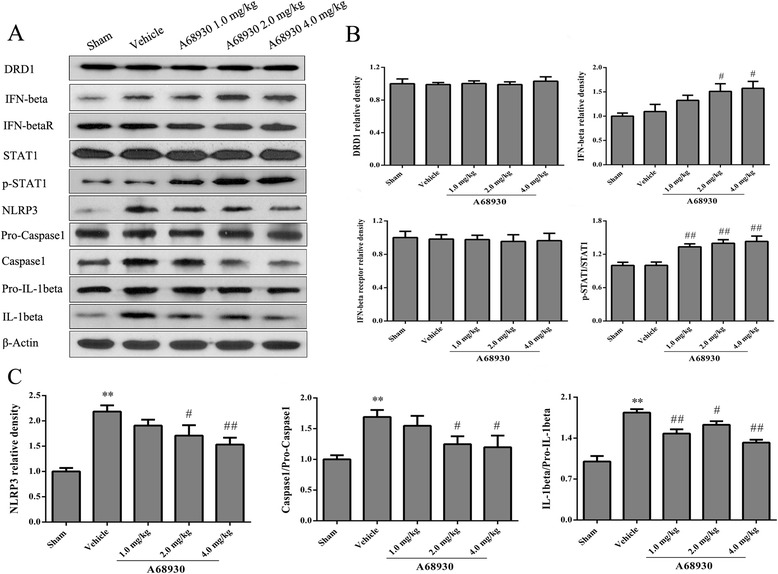
Effects of A68930 on the expressions of proteins in DRD1/IFN-beta/STAT1/NLRP3 pathway after ICH. **a** Representative photographs of the expressions of proteins in DRD1/IFN-beta/STAT1/NLRP3 pathway in western blotting. **b** and **c** Bar graphs of quantitative analysis of DRD1, IFN-beta, IFN-beta receptor, p-STAT1, NLRP3, pro-caspase1, caspase1, pro-IL-1beta, and IL-1beta, respectively. ^*^p < 0.05, ^**^p < 0.01 versus sham; ^#^*p* < 0.05, ^##^*p* < 0.01 versus vehicle

At 24 h after ICH, there were no changes in the expressions of DRD1 and IFN-beta receptor in the ipsilateral hemispheres. The expressions of NLRP3, caspase1, and IL-1beta were remarkably increased in vehicle group when compared to the sham group (*p* < 0.01; Fig. 5). However, A68930 treatment increased the expressions of IFN-beta, p-STAT1 and inhibited the expressions of NLRP3, caspase1, and IL-beta (*p* < 0.05 or *p* < 0.01 versus vehicle; Fig. 5).

### DRD1 antagonist and IFN-beta siRNA reversed effects of A68930 on neurological outcome and brain water content after ICH

**Fig. 6:**
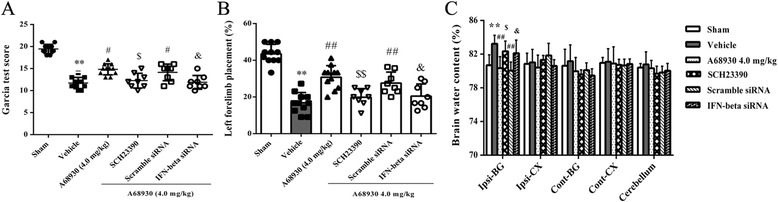
DRD1 antagonist and IFN-beta siRNA reversed effects of A68930 on neurological outcome and brain water content after ICH. Garcia test score (**a**), left forelimb placement (**b**), and brain water content (**c**) at 24 h after ICH. ^**^p < 0.01 versus sham; ^#^*p* < 0.05, ^##^*p* < 0.01 versus vehicle; ^$^*p* < 0.05, ^$$^*p* < 0.01 versus A68930 group; ^&^*p* < 0.05 versus scramble siRNA group

DRD1 antagonist administration and IFN-beta in vivo knockdown were performed to investigate the potential role of DRD1 and IFN-beta in the protective effects of A68930 in ICH mice. Pretreatment with DRD1 antagonist or IFN-beta siRNA reversed A68930-mediated improvement of neurological outcome in ICH mice as shown in Garcia test (*p* < 0.05; Fig. 6a) and limb placement test (*p* < 0.05 or *p* < 0.01; Fig. 6b). DRD1 antagonist or IFN-beta siRNA also abated A68930-mediated reduction of the brain water content in ICH mice (*p* < 0.05; Fig. 6c).

### DRD1 antagonist and IFN-beta siRNA reversed effects of A68930 on the expressions of proteins in DRD1/IFN-beta/NLRP3 pathway after ICH

**Fig. 7:**
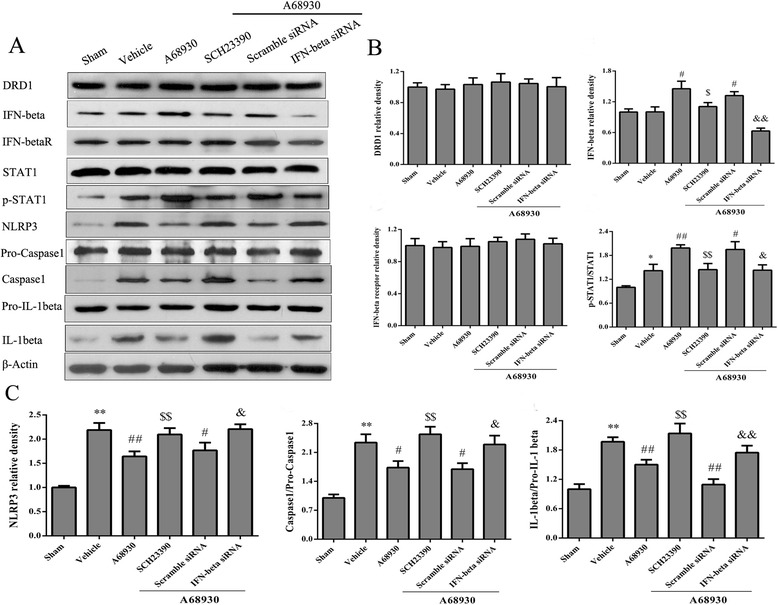
DRD1 antagonist and IFN-beta siRNA reversed effects of A68930 on DRD1/IFN-beta/STAT1/NLRP3 signaling pathway after ICH. **a** Representative photographs of the expressions of proteins in DRD1/IFN-beta/STAT1/NLRP3 pathway in western blotting. **b** and **c** Bar graphs of quantitative analysis of DRD1, IFN-beta, IFN-beta receptor, p-STAT1, NLRP3, pro-caspase1, caspase1, pro-IL-1beta, and IL-1beta, respectively. ^*^p < 0.05, ^**^p < 0.01 versus sham; ^#^*p* < 0.05, ^##^*p* < 0.01 versus vehicle; ^$^*p* < 0.05, ^$$^*p* < 0.01 versus A68930 group; ^&^*p* < 0.05, ^&&^*p* < 0.01 versus scramble siRNA group

To further confirm that DRD1 and IFN-beta were associated with the anti-inflammatory effect of A68930, the mice were subjected to DRD1 antagonist and IFN-beta siRNA. Then the expressions of protein in DRD1/IFN-beta/NLRP33 pathway were investigated in the ipsilateral hemispheres. It showed that pretreatment with DRD1 antagonist or IFN-beta siRNA decreased remarkably the expressions of IFN-beta and p-STAT1, while increased significantly the expressions of NLRP3, caspase1, and IL-1beta (*p* < 0.05 or *p* < 0.01; Fig. 7).

## Discussion

In the present study, we demonstrated that activation of DRD1 improved neurological outcome through inhibition of NLRP3-mediated inflammation in ICH mice. Specially, we found that activation of the DRD1 by A68930 decreased brain edema at 24 and 72 h after ICH, decreased the number of microglia and neutrophil in perihematomal areas at 24 h after ICH, increased IFN-beta and p-STAT1, and decreased NLRP3, caspase 1, and IL-1beta. In addition, DRD1 antagonist and IFN-beta siRNA reversed effects of A68930 on neurological outcome, brain edema, and protein expressions of DRD1/IFN-beta/NLRP3 pathway at 24 h after ICH.

Increasing evidences have shown that the immune system can be influenced by the nervous system and neurotransmitters [18]. The effect of dopamine and its agonists on immune responses including cytokine production has been reported [19]. Dopamine is found to suppress systemic inflammation and to inhibit the production of proinflammatory cytokines via DRD1 [8]. Dopamine can also attenuate inflammation in the post ischemic brain. Thus, levodopa treatment after experimental stroke reduced the infiltration of lymphocytes as well as the expression of endothelial intercellular adhesion molecule 1 in the ischemic brain [20]. Dopamine exerts its effects by binding to the dopamine receptors located on the surface of cells. There are at least five subtypes of dopamine receptors that have been identified, termed DRD1-DRD5, and most of them can be detected in immune cells, including macrophages and dendritic cells [21]. Previous results demonstrated that dopamine is an endogenous inhibitor of NLRP3 inflammasome activation, suggesting that dopamine is a potential anti-inflammatory chemical, in addition to a neurotransmitter. It also showed that DRD1 plays a primary role, while DRD2, DRD3, and DRD4 have no roles in dopamine-mediated NLRP3 inflammasome inhibition [11]. Consistent with those findings, our results demonstrated that A68930 activating DRD1 leads to the inhibition of inflammasome and then reduces the production of IL-1beta. Taken together, our findings indicated that dopamine may negatively regulate NLRP3 inflammasome activation via DRD1 signaling and suggested that DRD1 might be a potential target for treatment of ICH-induced inflammation.

In addition to its antiviral effects, IFN-beta is acknowledged as an immunomodulatory cytokine. IFN-beta has effects on the specific immune-mediated pathways. It is reported that IFN-beta affects antigen presentation, potentially shifts T lymphocytes polarization to a more anti-inflammatory state, then increases regulatory T-cell and B-cell activity, and reduces the ability of B cells to present antigens [22]. A recent study also demonstrated that IFN-beta inhibits IL-1beta production via the STAT1 transcription factor [12]. Phosphorylation of STAT1 represses the activity of the NLRP3 inflammasome, thereby suppressing caspase-1-dependent IL-1beta maturation. These results suggested that transcriptional induction of a target gene is required for the inflammasome-suppressing activity of IFN-beta. Our data showed that the ability of A68930 to suppress the inflammasome activity and the IL-1beta secretion may contribute to the activation of DRD1. In line with this, we showed that A68930 also results in the increase of p-STAT1 followed by the decrease of NLRP3 and caspase 1.

To further corroborate our hypothesis, SCH23390 or IFN-beta siRNA were administered before A68930 treatment. Consistent with our hypothesis, pretreatment with SCH23390 or IFN-beta siRNA reversed A68930-mediated improvement of neurological outcome and A68930-mediated decrease of brain edema after ICH. Furthermore, SCH23390 or IFN-beta siRNA also abolished the effect of A68930 on the expressions of p-STAT1, NLRP3, caspase 1, and IL-1beta. Therefore, it is reasonable to suggest that DRD1 activation-induced increase of IFN-beta may play a key role in improving neurological outcome through inhibition of NLRP3-mediated inflammation in ICH mice.

Several limitations exist in this study. Firstly, there are evidences indicating that microglia has two alternative activation phenotypes, termed the M1 phenotype and the alternative M2 phenotype. These different activation statuses of microglia are characterized by secretion of different cytokines [23]. The activation of M1 microglia is featured by the production of proinflammatory cytokines, e.g., IL-1beta and TNF-alpha, contributing to the amplification of the inflammatory responses during injuries. Conversely, M2 microglia plays an immunosuppressive role by antagonizing the classic M1 microglia and promoting tissue repair [24]. However, the present experiment did not distinguish the different activation forms of microglia. Secondly, the present study showed that A68930 increased the expression of IFN-beta and DRD1 specific antagonist reversed the augment of IFN-beta expression caused by A68930. However, the mechanisms of activation of DRD1 regulating the expression of IFN-beta are still unclear. Future studies will be needed to elucidate this signaling pathway.

## Conclusion

Collectively, our results demonstrated that activation of DRD1 with A68930 inhibits neuroinflammation and improves neurological outcome in ICH animal model, which is probably related to A68930-induced increase of IFN-beta followed by inhibiting the NLRP3-mediated inflammation. This novel observation indicates that although ICH injury can cause an inflammatory reaction of microglia, the IFN-beta in the brain can partly control the inflammation process. It also indicates that DRD1 agonists or IFN-beta have potentials to attenuate neuroinflammation and decrease secondary brain injury after ICH.

## Additional file

Additional file 1: Figure S1. Experimental design. ICH, intracerebral hemorrhage induced by bacterial collagenase infusion. (TIFF 1595 kb)

## Abbreviations

Cont-BG: Contralateral basal ganglia
Cont-CX: Contralateral cortex
DRD1: Dopamine D1 receptor
DRD2: Dopamine D2 receptor
Iba 1: Ionized calcium-binding adapter molecule 1
ICH: Intracerebral hemorrhage
IL-1beta: Interleukin-1beta
Ipsi-BG: Ipsilateral basal ganglia
Ipsi-CX: Ipsilateral cortex
