# Supplementary figures and images for: Activation of dopamine D1 receptor decreased NLRP3-mediated inflammation in intracerebral hemorrhage mice
Source: J Neuroinflammation. 2018 Jan 4;15:2. doi: 10.1186/s12974-017-1039-7 (PMC5753458; doi:10.1186/s12974-017-1039-7)

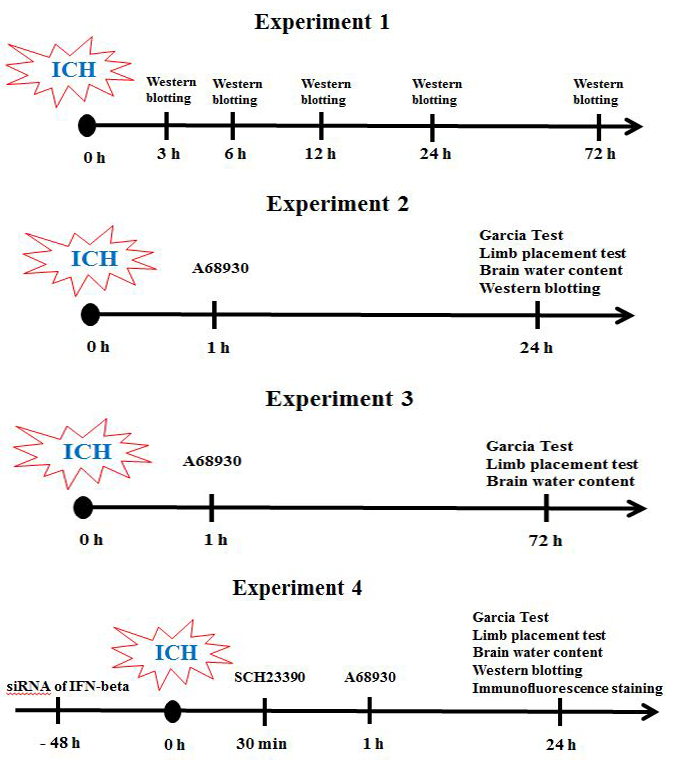

Supplement: Additional file 1: Figure S1. — Experimental design. ICH, intracerebral hemorrhage induced by bacterial collagenase infusion. (TIFF 1595 kb) [file 12974_2017_1039_MOESM1_ESM.tif]
